# Safety and immunogenicity of the COVID-19 vaccine BNT162b2 for patients with breast and gynecological cancer on active anticancer therapy: Results of a prospective observational study

**DOI:** 10.3389/fonc.2022.951026

**Published:** 2022-08-19

**Authors:** Pietro De Placido, Erica Pietroluongo, Carmine De Angelis, Margherita Tafuro, Chiara Barraco, Rosa Giannatiempo, Roberto Buonaiuto, Francesco Schettini, Anna Iervolino, Emilia Anna Vozzella, Mario Giuliano, Roberto Bianco, Grazia Arpino

**Affiliations:** ^1^ Department of Clinical Medicine and Surgery, University of Naples Federico II, Napoli, Campania, Italy; ^2^ Lester and Sue Smith Breast Center, Baylor College of Medicine, Houston, TX, United States; ^3^ Ospedale Evangelico Betania, Department of Pathology, Naples, Italy; ^4^ Medical Oncology Department, Hospital Clinic of Barcelona, Barcelona, Spain; ^5^ Faculty of Medicine, University of Barcelona, Barcelona, Spain; ^6^ Translational Genomics and Targeted Therapies in Solid Tumors, August Pi i Sunyer Biomedical Research Institute (IDIBAPS), Barcelona, Spain; ^7^ Direzione Generale, Azienda Ospedaliera Universitaria Federico II, Naples, Italy; ^8^ Direzione Sanitaria, Azienda Ospedaliera Universitaria Federico II, Naples, Italy

**Keywords:** COVID - 19, BNT162b2, COVID vaccine, breast cancer, chemotherapy, target therapies, immunogenicity, neutralizing antibody titers

## Abstract

**Background:**

Vaccines against severe acute respiratory syndrome coronavirus 2 (SARS-CoV-2) are highly effective. Nevertheless, immunocompromised participants were excluded from randomized controlled clinical trials. This study evaluates the efficacy and safety of the Pfizer/BioNTech BNT162b2 (BNT162b2) vaccine in patients with breast and gynecological cancer treated with active anticancer therapy versus a control cohort of healthy participants.

**Methods:**

Immune responses to the BNT162b2 vaccine in patients with breast cancer (*n* = 44) or a gynecological malignancy (*n* = 6) on active anticancer therapy (28 on chemotherapy, mostly anthracycline- or taxane-based, and 22 on target therapy) and in a control cohort of participants without cancer (*n* = 67) were investigated by SARS-CoV-2 neutralizing antibody titers measured by S1-binding immunoglobulin G (IgG) concentrations assessed using the LIAISON XL tools (DiaSorin S.p.A.). Response was assessed after a second dose of the BNT162b2 vaccine administered before and at least 3 weeks after the vaccine dose.

**Results:**

Overall, 43/50 (86%) patients of the cancer cohort (74% in the breast cancer group and 100% in the gynecological malignancy group) developed IgG antibodies after the second dose of the BNT162b2 vaccine. There were no statistically significant differences in responder rates between patients treated with chemotherapy and those on target therapy. The majority of patients who received chemotherapy with or without target therapy, 21/28 (75%), developed a reliable antibody titer after a vaccine. All seven non-responder patients were undergoing an anthracycline-based regimen. Based on IgG levels (0–400 AU/ml), patients were classified as negative (‘non-responders’), weakly positive, or strongly positive (‘responders’). No delay in cancer therapy schedule or reported side effects were recorded after BNT162b2 vaccine administration. All healthy participants were strongly positive. Responder rates differed significantly between the two study cohorts (p < 0.001).

**Conclusions:**

Most patients develop antibody titers after the second immunization. However, given the persistence of non-responders or weak responders, additional immunization booster seems to be required, along with proactive planning in the vaccination schedule, with vaccine administration spaced out over time with respect to chemotherapy.

## Introduction

In December 2019, a new acute respiratory syndrome, namely, coronavirus disease 2019 (COVID-19) caused by SARS-CoV-2 infection, emerged in the Chinese region of Wuhan, rapidly spread worldwide, and now has affected over 200 countries and territories (1). The World Health Organization (WHO) declared the state ‘pandemic’ on 11 March 2020 (2). Since then, more than 540 million (544,612,990) cases of COVID-19 have been confirmed up to June 2022, with 6 million (6,330,811) deaths (3).

The symptomatic severity of SARS-CoV-2 infection varies with age and comorbidities such as cancer, cardiovascular disease, diabetes, chronic respiratory diseases, and hypertension (4). Patients with cancer have a higher risk of SARS-CoV-2 infection and an increased risk of severe clinical events due to their weakened immune system caused by tumor growth or anticancer treatments (5).

A variety of anti-COVID-19 vaccines have been developed with unprecedented rapidity. Based on efficacy and safety data emerging from a large study conducted by Pfizer/BioNTech, their mRNA vaccine BNT162b2 was the first to be listed in the WHO Emergency Use Listing. This was on 31 December 2020 and was followed by SII/COVISHIELD and AstraZeneca/AZD1222, and Janssen/Ad26.COV 2.S Moderna COVID-19 vaccine (mRNA 1273) (6, 7). The first mass vaccination program started in early December 2020, and the number of vaccination doses administered is increasing daily. The latest count (June 2022) is 11,689,242,162 doses administered (8). On 22 December, the ‘*Agenzia Italiana del Farmaco*’ (AIFA) authorized one by one the marketing of four vaccines (Pfizer/BioNTech, Moderna, AstraZeneca, and Janssen) so that the Italian vaccination program could start aligned with other European countries (9).

According to the Italian Ministry of Health and AIFA guidelines, all frail patients, including cancer patients, are considered high-priority candidates to receive an mRNA-based vaccine, i.e., Pfizer/BioNTech or Moderna (10). However, clinical trials testing a vaccine’s efficacy largely excluded immunocompromised individuals, including patients on immunosuppressive therapies to control chronic inflammatory conditions, patients with primary immunodeficiencies, recipients of organ transplants, and patients with cancer on cytotoxic chemotherapy. Several recent reports have shown diminished immune responses to SARS-CoV-2 infections and mRNA vaccines in subsets of immunocompromised patients, although these vary greatly with the nature of the immunosuppressive therapy (11–14). Indeed, data on the preferred timing, effectiveness, and tolerability of a vaccine in cancer patients receiving active anticancer therapy are few and far between, and there are no data on the potential interference of immunosuppressive anticancer therapy on SARS-CoV-2 acquired immunity after one or more doses of vaccination.

The present study evaluates the tolerability and safety of BNT162b2 COVID-19 vaccination received while on active anticancer therapy in patients with breast and gynecological cancer and describes how the type of anticancer therapy received at the time of vaccination may affect the SARS-CoV-2 neutralizing antibody titers versus a control cohort of participants without cancer.

## Methods

### Study population

In the cancer cohort, 50 patients with breast or gynecological cancer on active anticancer therapy while receiving a dose of the BNT162b2 COVID-19 vaccine were prospectively enrolled in this study from May to October 2021 at the Oncology Unit of Federico II University Hospital. Forty-seven patients received the second vaccine dose and three the first dose at the study entrance. All cancer patients received the second vaccine dose 21 days later. A rapid antigenic test was performed in all cancer patients before each anticancer therapy cycle until February 2022.

According to international guidelines, the BNT162b2 COVID-19 vaccine was delivered in between anticancer therapy cycles and after appropriate waiting periods for patients receiving chemotherapy. In detail, the BNT162b2 COVID-19 vaccine was administered at least 3 days before or 3 days after anticancer therapy. SARS-CoV-2 neutralizing antibody titers were evaluated after each vaccination in all patients. Patients with previous COVID-19 infections were included in the study at the time they received vaccination according to local guidelines.

Information on clinical and tumor characteristics, type of anticancer therapy received, and adverse events after vaccination administration were collected for all the patients. In detail, patients were classified according to anticancer therapy into two groups: patients receiving chemotherapy (with or without target therapy) and patients receiving only target therapy. Patients were included in the anthracycline-based chemotherapy group if on epirubicin- or doxorubicin-based chemotherapy or had received anthracycline-based chemotherapy less than a month before the BNT162b2 mRNA vaccine.

The 67 participants in the control cohort of the observational study were all healthcare workers receiving the second dose of the BNT162b2 mRNA vaccine on day 21 after the first who consented to participate in the study as volunteers. None of them was assuming immunosuppressive therapy or had COVID-19 infection before receiving the vaccine.

The study was approved by the Institutional Review Board of the University of Naples Federico II protocol number 201/20, and all patients signed an informed consent form at the study entrance.

### Dosage of SARS-CoV-2 neutralizing antibody procedures

SARS-CoV-2 neutralizing antibody titers measured based on S1-binding immunoglobulin G (IgG) concentrations (15, 16) were available after vaccination for all cancer patients and healthy controls enrolled in the study. A peripheral venous blood sample drawn by an experienced and adequately personal protective equipment (PPE)-protected nurse was performed before and at least 3 weeks (21 ± 10 days) after the vaccine dose in our study population.

SARS-CoV-2 neutralizing antibody titers were executed using the LIAISON Xl tools DiaSorin S.p.A. using a UNI ISO 9001-2015 ITALCERT quality-certified laboratory according to the manufacturer’s instructions (16). According to IgG levels, patients were classified as non-responders if IgG levels were ≤25 AU/ml, weakly positive if IgG levels were between 25 and 100 AU/ml, and strongly positive if IgG levels were >100 AU/ml. Weakly and strongly positive patients were further classified as responders.

### Statistical methods

Descriptive statistics were used to describe patients’ demographics and tumor characteristics. Chi-squared with Yates’ correction statistics and Fisher’s exact test were used to compare distributions of categorical and continuous variables, respectively. Statistical significance was defined as a two-sided p-value <0.05.

## Results

### Demographics

Fifty patients in the cancer cohort, 44 with breast and six with gynecological cancer, were prospectively enrolled in the present study from May to October 2021 at the Oncology Unit of the Federico II University Hospital. **Table 1** shows the patient population included in this study. The median age was 55 years (range between 29 and 88 years) in the overall population. Patients with gynecological cancer were slightly older with a median age of 60 years. Most patients (96%) had an Eastern Cooperative Oncology Group (ECOG) Performance Status (PS) between 0 and 1. Of the 44 breast cancers, 23/44 (52%), 18/44 (41%), and 3/44 (7%) were luminal, HER2-positive (HER2+), and triple-negative (TN) breast cancers. At study entry, 19/44 (40%) and 25/44 (60%) patients had early and metastatic disease, respectively. All patients with early breast cancer received sequential chemotherapy with four cycles of either epirubicin or doxorubicin plus cyclophosphamide (EC or AC, respectively) every 15 or 21 days followed by weekly paclitaxel (P) for 12 weeks. According to current guidelines, while on adjuvant EC, all eligible patients received granulocyte colony-stimulating factor support. Anti-HER2 therapy was added in case of HER2 positivity, defined by immunohistochemistry (IHC) and/or fluorescence *in situ* hybridization (FISH) for HER2, in either the adjuvant or neoadjuvant setting. Among patients with early breast cancer, 10 were receiving EC or AC, five were receiving P with anti-HER2 therapy if HER2-positive, and four were receiving anti-HER2 target alone at the time of the second dose of the BNT162b2 COVID-19 vaccine. Among patients with advanced breast cancer, nine and 16 had chemotherapy (with or without target agents) and target agents alone, respectively, at the time of the BNT162b2 COVID-19 vaccine (**Table 1**).

**Table 1:** Patients’ characteristics.

***Total number of patients***	50
**ECOG**	
Ø	31(62%))
1	17 (34%)
2	2(4%)
***Median age***	**55 (28–86)**
≤50 years	19 (38%)
>50 years	31(62%)
***Sex***	
Male	1/50 (2%)
Female	49/50 (98%)
***Breast cancer***	
Number of patients	44 (88%)
Histotype	
Luminal	23 (46%)
HER2+	18 (36%)
Triple negative	3 (6%)
Stages	
I–III	19/44 (38%)
IV	25/44 (50%)
***Gynecological cancer***	
Number of patients	6 (12%)
Stages	2/6 (33%)
I–II	4/6 (77%)
IV	
**Breast cancer patients**	
Number of patients	**44 (88%)**
•**Early breast cancer setting**	**19/44 (38%)**
Anthracycline-based chemo± target therapy*	15
Maintenance target therapy without chemo*	4
• **Advanced breast cancer**	**25/44 (50%)**
Chemotherapy ± target therapy*	9
Target therapy without chemo**	16
**Gynecological tumors**	
Number of patients	**6/50 (12%)**
•**Early setting**	2/6 (33%)
Platinum-based chemotherapy	2
•Advanced disease	4/6 (77%)
Platinum-based chemo	2
Target therapy without chemo***	2

Chemotherapies: single agents platinum (2 pts), eribulin (2 pts), and paclitaxel (5 pts) monochemotherapy.

target therapies: * pertuzumab and trastuzumab or trastuzumab alone; ** pertuzumab plus trastuzumab or trastuzumab alone, TDM1, bevacizumab; *** bevacizumab.

ECOG, Eastern Cooperative Oncology Group.

Among gynecological cancer patients, 2/6 (33%) and 4/6 (77%) patients had early and metastatic disease, respectively. In the early setting, both patient groups were receiving a platinum-based chemotherapy regimen. In the advanced setting, one patient was on platinum-based chemotherapy and two patients were on target therapy (bevacizumab) without chemotherapy at the time of the second dose of the BNT162b2 COVID-19 vaccine. Participants of the control group were younger than those of the cancer cohort (median age = 31 years, range 26–68). No relevant comorbidities were recorded for these participants.

### Vaccine effectiveness and safety

In our cohort of cancer patients, three had a previous SARS-CoV-2 diagnosis; however, SARS-CoV-2 neutralizing antibody titers were weak or not dosable for all of them at the time of BNT162b2 COVID-19 vaccine administration.

Overall, 43/50 (86%) patients of the cancer cohort, 37/44 (74%) in the breast cancer, and 6/6 (100%) in the gynecological cancer group developed IgG after the second dose of the BNT162b2 COVID-19 vaccine (**Table 2**). There was no statistically significant difference in responder rates between the chemotherapy and targeted therapy-only treated patients. In detail, 21/50 (42%) chemotherapy (with or without target therapy) patients *vs* 22/50 (44%) target therapy-only patients were classified as ‘responders’ (p = 0.56). Taking into account chemotherapy-treated patients (with or without target therapy), the vast majority of them, 21/28 (75%), developed a reliable antibody titer after the BNT162b2 COVID-19 vaccine.

**Table 2:** BNT162b2 COVID-19 vaccine responder rates according to anticancer treatment.

Type of tumor	Responders	Non-responders
	43 (86%)	7 (14%)
Breast cancer	37 (74%)	7 (14%)
•Therapy in an early setting	12 (24%)	7 (14%)
Anthracycline-based chemotherapy	8 (16%)	7 (10%)
Target therapy	4 (8%)	0 (0%)
•Therapy for advanced disease	25 (50%)	0 (0%)
Anthracycline-based chemotherapy	0 (0%)	0 (0%)
Non-anthracycline-based chemotherapy	9 (18%)	0 (0%)
Target therapy	16 (32%)	0 (0%)
Gynecological tumor	6 (12%)	0 (0%)
•Therapy in early setting	2 (4%)	0 (0%)
• Non-anthracycline-based chemotherapy	2 (4%)	0 (0%)
•Therapy for advanced disease	4 (8%)	0 (0%)
• Non-anthracycline-based chemotherapy	2 (4%)	0 (0%)
Target therapy	2 (4%)	0 (0%)

**Figure 1:**
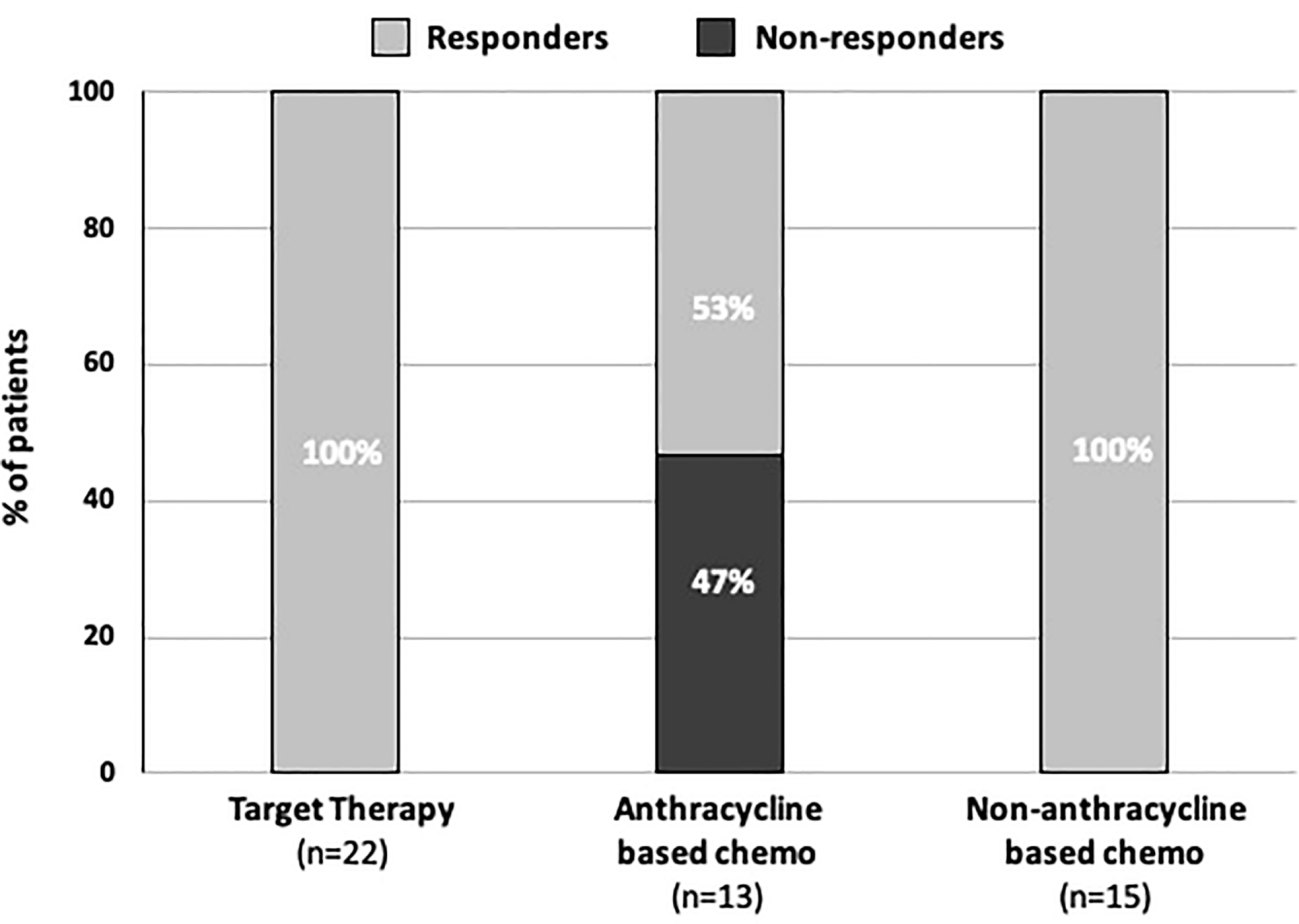
Bar graphs showing the seroconversion rate according to treatment.

Interestingly, all seven non-responder patients were undergoing an anthracycline-based chemotherapy regimen for breast cancer in the neo/adjuvant setting: five on adjuvant EC/AC and two patients on adjuvant P shortly after four cycles of EC/AC with or without target therapy. Overall, patients on EC/AC or who received EC/AC shortly before the BNT162b2 COVID-19 vaccine were more likely to be non-responders than all the other patients. In fact, seven non-responder patients in the anthracycline-based chemotherapy group *vs* zero in all other therapies group were observed. Conversely, responders constituted only 8/15 (53%) in the anthracycline-based regimen group *vs* 35/35 (100%) in all other therapies group (chemotherapy, chemotherapy plus target therapy, or target therapy alone) (p < 0.001) (**Table 3**) (**Figure 1**).

**Table 3:** Responder rates in anthracycline-based regimen *vs* all other therapies.

Therapy	Responders	Non-Responders	p-Value (chi-square test)
***Anthracycline-based regimen***	8 (53%)	7 (47%)	p < 0.001
All other therapies	35 (100%)	0	

Among responder cancer patients, considering the antibody titers, 28 (56%) and 15 (30%) were strongly and weakly positive, respectively (**Table 4**). In detail, 13/21 (62%) and 8/21 (38%) patients in the 21 chemo-treated responders and 15/22 (68%) *vs* 7/22 (32%) in the 22 target-only responders were classified as strong and weak responders, respectively. Among responders, there was no statistically significant difference in antibody titer according to therapy received before the BNT162b2 COVID-19 vaccine.

**Table 4:** SARS-CoV-2 neutralizing antibody titers according to anticancer therapy.

	Strongly positive	Weakly positive	Serum negative
All cancers	**28 (56%)**	**15 (30%)**	**7 (14%)**
•Therapy in early BC setting	**7 (14%)**	**5 (10%)**	**7 (14%)**
Anthracycline-based chemotherapy	5 (10%)	3 (6%)	7 (14%)
*Target therapy*	2 (4%)	2 (4%)	0 (0%)
•Therapy for advanced BC disease	**16 (32%)**	**9 (18%)**	**0 (0%)**
*Non-anthracycline-based chemotherapy*	5 (10%)	4 (8%)	0 (0%)
*Target therapy*	11 (22%)	5 (10%)	0 (0%
* ***Therapy in early GC setting***	**2 (4%)**	**0 (0%)**	**0 (0%)**
* *Non-anthracycline-based chemotherapy*	2 (4%)	0 (0%)	0 (0%)
* ***Therapy for advanced GC disease***	**3 (6%)**	**1 (2%)**	**0 (0%)**
*Non-anthracycline-based chemotherapy*	1 (2%)	1 (2%)	0 (0%)
* *Target therapy*	2 (4%)	0 (0%)	0 (0%)

BC, breast cancer; GC, gynecological cancer.

In the early breast cancer patients’ group, of the 12 responders, the 8/12 weakly positive patients were receiving anthracycline-based chemotherapy, and the 4/12 strongly positive patients were receiving maintenance anti-HER2 therapy at the time of the BNT162b2 COVID-19 vaccine. However, anthracycline-based chemotherapy was not statistically associated with a weaker IgG response than maintenance therapy in this setting. Both patients receiving adjuvant therapy for gynecological cancer were strongly positive.

All the patients with either breast or gynecological advanced disease were responders: 19 (38%) and 10 (20%) were strongly positive and weakly positive, respectively, regardless of whether they received chemotherapy at the time of the BNT162b2 COVID-19 vaccine. Notably, none of the advanced disease patients have had anthracycline-based regimens or polychemotherapy during or shortly before starting the COVID-19 vaccination program. Overall, no cases of symptomatic or asymptomatic SARS-CoV-2 infection were reported at 4 months of follow-up in the whole cancer patient cohort.

Regarding safety, no delay in cancer therapy schedule or side effects were recorded after BNT162b2 COVID-19 vaccine administration. All 67 participants of the control cohort seroconverted after the BNT162b2 COVID-19 vaccine. All healthy participants became strongly positive responders. Responder rates differed significantly between the two study cohorts (p < 0.001) as well as rates of weak and strong positivity among responders (p < 0.001) (**Table 5**).

**Table 5:** SARS-CoV-2 neutralizing antibody titers among cancer patients and healthy workers.

	Strongly positive	Weakly positive	Serum negative	p-Value
***Cancer patients***	28 (56%)	15 (30%)	7 (14%)	p < 0.001
Health workers	67 (100%)	0	0	

## Discussion

Given the increased mortality rate of up to 13% of patients with cancer and SARS-CoV-2 infection and the worrisome complications including delays in cancer treatment, the National Comprehensive Cancer Network and other oncologic societies, such as the American Society of Clinical Oncology and European Society for Medical Oncology, have recommended the SARS-CoV-2 vaccine to patients with active cancer regardless of therapy (17–20).

Multiple cohorts have now been examined, mainly in patients with hematologic disease, and they all suggest that patients with cancer do not mount the same antibody responses to the BNT162b2 vaccine as healthy controls and could still develop SARS-CoV-2 infection with severe and critical symptoms (21). Similar studies also show the reduced mRNA vaccine immunogenicity in patients receiving immunosuppressive medications, such as rituximab or mycophenolate, and in patients with liquid tumors (22). However, similar data on solid cancer patients are still sparse and far from conclusive.

In agreement with previous findings reported in patients with solid cancer (14, 23), we observed overall lower antibody levels in patients with cancer versus control cohorts. However, the rates of seroconversion were higher in our patient population than in those included in other studies (24, 25). Differences may be mainly due to different patient populations and types of therapy delivered. In our dataset, we enrolled patients receiving the second vaccination dose, we did not enroll patients on immunotherapy or with hematologic malignancies, and, importantly, we also included patients on target biological therapy only, which is obviously less immunosuppressive than chemotherapy. Interestingly, not all chemotherapy regimens seem to negatively affect antibody production to the same extent. National guidelines discourage cyclophosphamide administration on the edge of vaccination (26), even if recent data on hematological patients undergoing allogeneic stem cell transplantation showed that the use of cyclophosphamide conditioning did not impair significantly patients’ seroconversion (27, 28). In our cancer cohort, patients receiving anthracycline-based regimens, either dose dense or not, had a higher impact on antibody production than non-anthracycline regimens. Interestingly, anthracyclines were delivered with cyclophosphamide in all our cases. Of note, all non-responders to vaccination were breast cancer patients receiving anthracyclines and cyclophosphamide as adjuvant chemotherapy regimens. However, though a formal analysis was not performed due to the small dataset, we speculate that the higher rates of vaccination failure were mainly due to the type of chemotherapy delivered in these patients (all had anthracycline-based regimens) rather than to tumor histotype or stage.

Safety SARS-CoV-2 mRNA vaccines among cancer patients have been the object of several studies worldwide (14, 23). Most of the reported adverse events are arm pain and fatigue, usually grade 1 or 2 (11, 29–32). Less than 5% of patients report grade 3 or worst adverse events (33). No relevant adverse events or anticancer therapy delay was observed in our metastatic disease cohort of patients. Importantly, no delay in the vaccination schedule was necessary for our patients; thus, the second vaccine dose was performed as indicated 21 days after the first dose.

Although correlates of protection against SARS-CoV-2 are not well defined, vaccine immunogenicity is broadly assumed to require neutralizing antibodies and antigen-specific T cells. A major limitation of our study is the lack of data on cellular immune responses. However, there are no approved tests with which to assess the T cell-mediated immune and protective response against SARS-CoV-2. Irrespective of immunogenicity data, it could be argued that evidence of high vaccine efficacy, eventually mediated by T-cell response, in our study population, is provided by the fact that there were no new positive antigenic tests 21 days after vaccination and that none of the cancer study cohorts developed symptomatic or asymptomatic SARS-CoV-2 infection in the 4 months following study enrollment. Another important limitation is the lack of information about antibody titer before the second dose of the vaccine. Recent data suggest that most patients with cancer on active chemotherapy are likely to have impaired antibody levels, which may not protect patients from the disease after the second or third immunization (14). Despite the booster vaccine dose, some of our patients developed a weak or null response to the second immunization. Consequently, more immunizations or other strategies are needed to protect from SARS-CoV-2 in this subset of patients. Recent studies showed that irrespective of the administration of additional vaccine boosts in patients receiving active anticancer therapies, increases in antibodies and recalcitrance of T cells are still modest as compared to controls (13, 23, 34). Therefore, expectations should remain tempered as to the degree of benefit in this setting, and in the future, quantitative antibody tests can potentially be used to select individuals who need and would benefit the most from a booster.

Although our sample size was too small to draw definitive conclusions, the prospective nature of this study, together with the inclusion of only breast and gynecological cancers plus the lack of heterogeneity as regards the timing of cancer therapies with respect to vaccine dose, means that our findings are reliable. In this perspective, our study is hypothesis-generating, and the results merit further exploration in larger cohorts to better understand the ideal timing for vaccination in patients on active immunosuppressive therapy. All participants in the control group developed a high antibody titer after the second dose of vaccine, and when compared to individuals not on anticancer therapy, the magnitudes of vaccine-induced antibodies were substantially reduced in patients with cancer. Notably, controls were much younger than cancer patients, and this may affect the magnitude of the differences. However, not all anticancer regimens impaired neutralizing antibody responses to the same levels. It is worth noting that all our patients, responders and not, underwent blood collection for immune analysis before receiving the next chemotherapy cycle; thus, none of them was immunosuppressed. If feasible, adjustment of vaccine schedule before starting chemotherapy may help to ensure optimal immune response in this subgroup.

Taken together, our data suggest that most patients with cancer on active therapy are likely to have antibody levels, which have been correlated with protection against the disease, after the second immunization. However, given the persistence of non-responders or weak responders, especially among polychemotherapy-treated patients, an additional immunization booster, proactive planning in schedule vaccine administration, with vaccine administration spaced out over time with respect to chemotherapy, or different strategies may be needed in these patients.

## Data availability statement

The raw data supporting the conclusions of this article will be made available by the authors, without undue reservation.

## Ethics statement

This study was reviewed and approved by Institutional Review Board of the University of Naples Federico II. The patients/participants provided their written informed consent to participate in this study.

## Author contributions

All authors contributed to the article and approved the submitted version.

## Conflict of interest

CD reports personal fees from Roche, AstraZeneca, Lilly, GSK, Novartis, Seagen and Pfizer, Advisory Board for Roche, AstraZeneca, Lilly, GSK, Novartis, Seagen and Pfizer, support for attending meetings and/or travel Roche, AstraZeneca, Lilly, GSK, Novartis, Celgene and Pfizer, grants from Novartis. FS received a European Society for Medical Oncology (ESMO) Fellowship -Translational and the 2021 BBVA Foundation/Hospital Clinic of Barcelona Joan Rodés -Jose Baselga Advanced Research Contract in Oncology. MG reports consulting or advisory Role from Lilly, Seagen, Roche, MSD, Gilead, Novartis and Pfizer, Speaker’s Bureau for Lilly, Seagen, AstraZeneca, Novartis and Pfizer, support for attending meetings and/or travel Novartis and Pfizer, Roche, grants from Novartis. RBi is on the Advisory Board for Pfizer, Astrazeneca and Novartis; GA reports personal fees from Novartis, during the conduct of the study; personal fees from Lilly, grants and personal fees from Roche, grants, personal fees and non-financial support from Pfizer, grants, personal fees and non-financial support from AstraZeneca, personal fees from Daichi, outside the submitted work.

The remaining authors declare that the research was conducted in the absence of any commercial or financial relationships that could be construed as a potential conflict of interest.

The reviewer UM declared a shared affiliation with the authors to the handling editor at time of review.

## Publisher’s note

All claims expressed in this article are solely those of the authors and do not necessarily represent those of their affiliated organizations, or those of the publisher, the editors and the reviewers. Any product that may be evaluated in this article, or claim that may be made by its manufacturer, is not guaranteed or endorsed by the publisher.
